# Malignant pleural mesothelioma in a young adult without asbestos exposure: a case report highlighting diagnostic pitfalls and literature review

**DOI:** 10.1097/MS9.0000000000005011

**Published:** 2026-04-15

**Authors:** Rohan Chauhan, Kashish Fnu, Bhavna Sahil, Arshnoor Kaur Chug, Mirza Mohammad Ali Baig, Muhammad Umar, Iman Osman Abufatima

**Affiliations:** a Department of Medicine, Fortis Hospital Ludhiana, Ludhiana, Punjab, India; b Department of Medicine, Christian Medical College and Hospital, Ludhiana, Punjab, India; c Department of Medicine, Shri Guru Ramdas Charitable Hospital, Amritsar, Punjab, India; d Department of Medicine, Islamic International Medical College, Riphah International University, Rawalpindi, Pakistan; e Department of Medicine, Khairpur Medical College, Khairpur Mir’s, Pakistan; f Department of Medicine, University of Medical Sciences and Technology, Khartoum, Sudan

**Keywords:** BAP1, immunohistochemistry, malignant pleural mesothelioma, pleural effusion, young adult

## Abstract

**Background:**

Malignant pleural mesothelioma (MPM) is a rare and aggressive malignancy typically associated with asbestos exposure in older adults. Its occurrence in young individuals without asbestos exposure poses significant diagnostic challenges, particularly in tuberculosis-endemic regions.

**Case presentation:**

We report the case of a 23-year-old man with no history of asbestos exposure who presented with progressive pleuritic chest pain, dyspnea, fever, and weight loss. Initial evaluation suggested tuberculous pleuritis, and empirical therapy was initiated. Imaging revealed diffuse nodular pleural thickening with loculated effusion. Ultrasound-guided pleural biopsy demonstrated epithelioid MPM, confirmed by immunohistochemical positivity for calretinin and WT-1 with TTF-1 negativity. Despite supportive care, the patient experienced rapid clinical deterioration and died within 1 week of histopathological confirmation. Genetic testing for BAP1 mutation was recommended but could not be performed due to rapid disease progression.

**Conclusion:**

This case underscores the importance of considering MPM in the differential diagnosis of pleural effusion in young adults, even in the absence of asbestos exposure. Early pleural biopsy and immunohistochemical evaluation are essential to avoid diagnostic delay, particularly in tuberculosis-endemic settings.

## Introduction

Malignant pleural mesothelioma (MPM) is a rare and highly aggressive malignancy arising from the mesothelial lining of the pleura, most commonly associated with asbestos exposure and typically diagnosed in older adults^[^1,2^]^. Despite advances in diagnostic techniques and multimodality treatment, the prognosis remains poor, with reported median survival ranging from 9 to 18 months^[^2^]^.

The occurrence of MPM in children, adolescents, and young adults is exceedingly uncommon, particularly in the absence of documented asbestos exposure^[^3–5^]^. Consequently, young-onset, asbestos-negative MPM remains under-represented in the literature, and its clinical behavior, optimal diagnostic approach, and prognostic determinants are not well defined. Emerging evidence suggests that this subgroup may represent a biologically distinct entity, with genetic susceptibility playing a more prominent role than environmental exposure alone^[^6,7^]^.

**HIGHLIGHTS**

- Young-onset mesothelioma can occur without asbestos exposure.
- Often misdiagnosed as tuberculosis in endemic regions.
- Immunohistochemistry confirms epithelioid mesothelioma.
- Possible link to germline BAP1 mutation.
- Early biopsy is key to timely diagnosis.

In tuberculosis (TB)-endemic regions, the diagnosis of MPM is further complicated by the high prevalence of tuberculous pleuritis. Young patients presenting with pleural effusion, systemic symptoms, and radiologic abnormalities are frequently managed empirically for TB, often without early histopathological confirmation^[^4,8^]^. This diagnostic overlap can result in significant delays in identifying underlying malignancy, leading to advanced disease at presentation and limited therapeutic options.

Reporting cases of young-onset, asbestos-negative MPM from TB-endemic settings is therefore clinically important, as it highlights critical diagnostic pitfalls and reinforces the need for early pleural biopsy in atypical or non-resolving pleural effusions. We present a case of rapidly progressive MPM in a young adult without asbestos exposure, illustrating the diagnostic challenges encountered in a TB-endemic region and placing the findings in the context of existing literature.

## Case presentation

A 23-year-old man from rural Bihar, India, with no prior medical illness, asbestos exposure, or family history of malignancy, presented with a 4-month history of persistent right-sided lumbar and pleuritic chest pain. These symptoms were accompanied by progressive exertional dyspnea, intermittent low-grade fever, anorexia, generalized weakness, and a weight loss of approximately 1.5 kg.

On admission, the patient was alert, oriented, and hemodynamically stable. Respiratory examination revealed markedly reduced air entry over the right infrascapular region, while cardiovascular and abdominal examinations were unremarkable. A concise timeline of the patient’s clinical course is summarized in Table 1, outlining the progression from symptom onset to diagnosis and outcome.

**Table 1:** Concise clinical timeline of disease course and outcome.

Time point	Key events and findings
~4 months prior	Progressive right-sided chest and lumbar pain, dyspnea, intermittent fever, anorexia, and ~1.5 kg weight loss
Day 1	Admission; decreased right infrascapular air entry; CBC: anemia, leukocytosis, thrombocytosis; and ultrasound chest: right pleural effusion (~45 mL) with lung collapse
Day 2–3	HRCT chest: loculated right pleural effusion; CECT chest: diffusely thickened enhancing pleura with smooth undulating margins, minimal fluid; neoplastic etiology suspected
Day 4–5	Ultrasound-guided pleural tap attempted (dry tap); pleural biopsy performed
Day 7	Histopathology and IHC confirmed epithelioid malignant pleural mesothelioma (calretinin+, WT-1+, and TTF-1−)
Days 7–8	Systemic therapy discussed; germline BAP1 testing recommended (not performed)
Days 8–10	Rapid clinical deterioration with hypoxemic respiratory failure; death within 48 hours of deterioration onset

**Figure 1.**
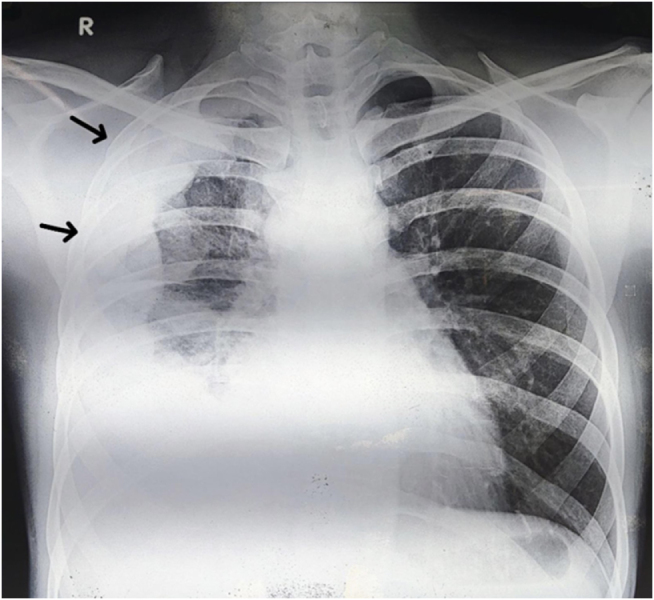
Chest radiograph demonstrating a right-sided pleural effusion with associated ipsilateral volume loss and irregular pleural margins (black arrows), raising suspicion for pleural-based pathology.

**Figure 2.**
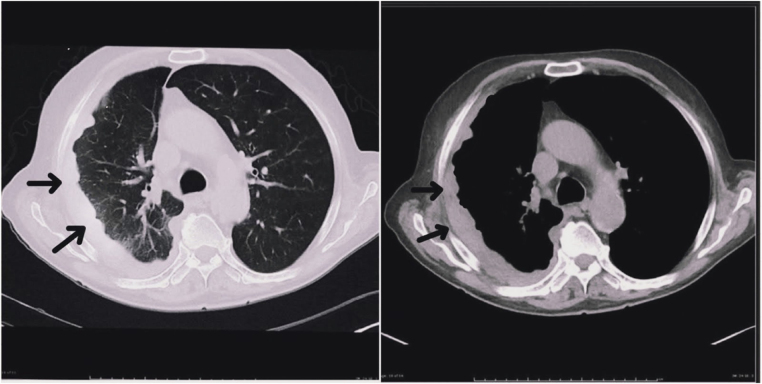
High-resolution and contrast-enhanced computed tomography of the chest showing diffuse nodular and rind-like thickening of the right pleura with smooth undulating margins (black arrows), minimal residual pleural fluid, and underlying lung compression, characteristic of malignant pleural mesothelioma.

Laboratory investigations revealed normocytic anemia, leukocytosis, reactive thrombocytosis, and elevated inflammatory markers. Chest radiography demonstrated a right-sided pleural effusion with associated volume loss (Fig. 1). Thoracic ultrasound showed a loculated pleural collection, and contrast-enhanced computed tomography (CECT) of the chest revealed diffuse nodular pleural thickening with basal atelectasis and ipsilateral volume loss, without mediastinal invasion (Fig. 2).

CECT of the abdomen was normal, with no evidence of intra-abdominal malignancy, lymphadenopathy, or peritoneal involvement.

**Figure 3.**
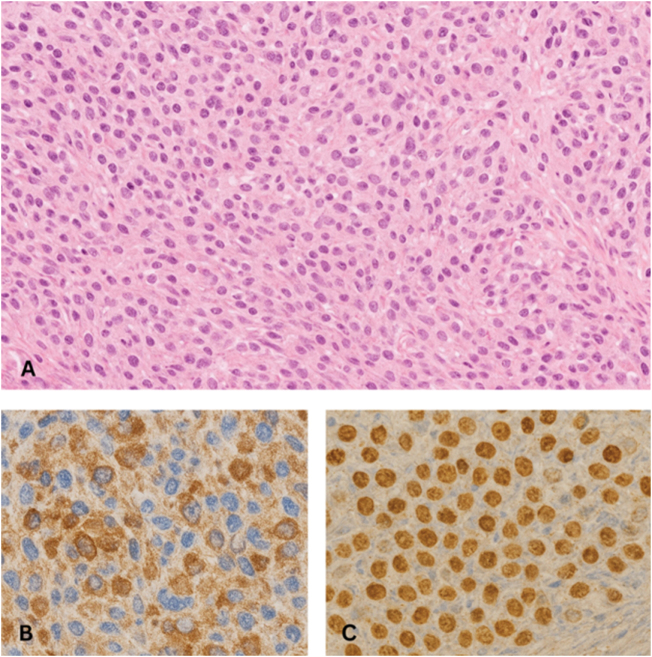
(A) Hematoxylin and eosin–stained section showing epithelioid tumor cells arranged in cords and nests infiltrating fibroconnective tissue, with moderate eosinophilic cytoplasm and mild nuclear pleomorphism. (B) Calretinin immunostain demonstrating strong nuclear and cytoplasmic positivity in tumor cells. (C) WT-1 immunostain showing diffuse nuclear positivity, supporting mesothelial differentiation.

An ultrasound-guided pleural aspiration was attempted but resulted in a dry tap, consistent with a loculated pleural process. Subsequently, an ultrasound-guided pleural biopsy was performed. Histopathological examination demonstrated epithelioid tumor cells arranged in cords and nests, infiltrating the fibroconnective tissue. Immunohistochemistry (IHC) showed strong nuclear and cytoplasmic positivity for calretinin and WT-1, with negativity for TTF-1, supporting the diagnosis of epithelioid MPM (Fig. 3).

Given the patient’s young age and absence of occupational or environmental asbestos exposure, germline BAP1 testing was recommended because of its well-established association with early-onset, asbestos-negative MPM and its potential implications for familial risk assessment and screening. However, genetic testing could not be performed due to financial constraints and the patient’s rapid clinical deterioration.

Despite supportive management, the patient developed progressive hypoxemic respiratory failure attributable to extensive pleural disease and died approximately 1 week after histopathological confirmation.

## Discussion

MPM is an uncommon and highly aggressive malignancy, classically associated with asbestos exposure and typically diagnosed in older adults^[^1,2^]^. The occurrence of MPM in young individuals without a history of asbestos exposure is exceedingly rare and presents distinct diagnostic challenges. In TB-endemic regions, pleural effusion in young patients is frequently attributed to tuberculous pleuritis, often leading to empiric anti-tubercular therapy without early histological confirmation. As demonstrated in the present case, such an approach can delay recognition of an underlying malignancy and adversely affect clinical outcomes^[^4,5^]^.

Although IHC is not absolutely specific when interpreted in isolation, contemporary diagnostic standards emphasize its integration with clinical presentation, radiologic findings, and histomorphology. In our patient, the diagnosis of MPM was established based on a concordant triad of clinical features, characteristic pleural-based imaging abnormalities, and epithelioid histology supported by a mesothelioma-specific immunohistochemical profile. Strong nuclear and cytoplasmic positivity for calretinin and WT-1, together with TTF-1 negativity, is widely accepted as a reliable marker combination for mesothelial differentiation when interpreted in the appropriate clinicopathologic context^[^1,2^]^. While an expanded IHC panel including CK5/6, BerEP4, CEA, and CK20 may provide additional exclusionary value, current classification frameworks recognize that a limited but targeted panel is sufficient when supported by compatible morphologic and radiologic findings. Furthermore, the absence of an extra-thoracic primary malignancy on abdomino-pelvic imaging in our patient strengthened diagnostic certainty.

Published reports confirm that MPM in young, asbestos-negative individuals is exceptionally rare and exhibits marked clinical heterogeneity. Montoya *et al* described a 22-year-old healthy man with epithelioid MPM who survived beyond 12 months following chemotherapy^[^5^]^. In contrast, Chem *et al* reported a 17-year-old boy who died within 4 months of diagnosis despite similar histology^[^6^]^. In a larger clinicopathologic and genetic analysis of young patients with pleural diffuse mesothelioma, Hung *et al* reported a median overall survival of approximately 11 months^[^9^]^. Compared with these reports, our patient experienced rapid clinical deterioration and death within 1 week of histopathological confirmation, representing one of the most fulminant disease courses reported to date despite epithelioid histology.

The striking variability in outcomes among young patients underscores that prognosis in MPM is influenced by multiple factors beyond histologic subtype alone. In the present case, advanced pleural disease burden at presentation, diagnostic delay related to initial empiric anti-tubercular therapy, and limited opportunity for timely oncologic intervention likely contributed to the poor outcome. These factors may outweigh the traditionally favorable prognostic implications of epithelioid morphology and highlight the critical importance of early tissue diagnosis in atypical pleural effusions, particularly in high TB-burden settings^[^4,8^]^.

Genetic susceptibility has emerged as an important consideration in young-onset, asbestos-negative mesothelioma. Recent studies have demonstrated that germline mutations, particularly involving the BAP1 gene and related pathways, predispose individuals to early-onset mesothelioma and may influence disease behavior^[^7,9^]^. Although genetic testing could not be performed in this patient due to financial constraints and rapid clinical decline, the young age at presentation and aggressive clinical course raise the possibility of an underlying genetic predisposition.

Epidemiologic evidence further supports the existence of mesothelioma in young populations as a distinct clinical entity not fully explained by asbestos exposure. Yeung *et al* demonstrated that mesothelioma incidence in young individuals, while rare, is real and cannot be entirely attributed to occupational exposure^[^10^]^. Additionally, audits from resource-limited settings have highlighted persistent diagnostic delays and poorer outcomes, reflecting disparities in access to early diagnostic and therapeutic interventions^[^8^]^.

A comparative summary of reported young-onset, asbestos-negative MPM cases is presented in Table 2. While survival outcomes vary considerably across studies, the present case is notable for its exceptionally rapid progression, emphasizing the potential consequences of diagnostic delay and advanced disease at presentation.

**Table 2:** Comparative summary of reported cases of young-onset, asbestos-negative malignant pleural mesothelioma.

Author/year	Age/sex	Exposure	Histology	Outcome
Khatib *et al*, 2021^[^5^]^	22 M	None	Epithelioid	Alive at 12 months (chemo)
Pérez-Guzmán *et al*, 2016^[^6^]^	17 M	None	Epithelioid	Died at 4 months
Vivero *et al*, 2018^[^9^]^	<35 years cohort	Variable	Mixed	Median OS 11 months
Marinaccio *et al*, 2023^[^10^]^	Young cohort	Variable	Mixed	Epidemiologic evidence of incidence
Present case	23 M	None	Epithelioid	Death within 1 week

Overall, this case highlights several important clinical lessons: MPM should remain a differential diagnosis in young patients presenting with unexplained pleural effusion, even in the absence of asbestos exposure; early pleural biopsy and immunohistochemical evaluation are essential to avoid diagnostic delay; and consideration of underlying genetic susceptibility is increasingly relevant in this patient population.

## Limitations

This case report has several limitations. An expanded immunohistochemical panel and germline genetic testing, including BAP1 analysis, could not be performed due to resource constraints and the patient’s rapid clinical deterioration. Pleural fluid biochemical analysis was not available because ultrasound-guided aspiration resulted in a dry tap, and no autopsy was performed to assess the full extent of the disease. Despite these limitations, the diagnosis was established using a concordant clinicopathologic approach supported by characteristic imaging findings and mesothelioma-specific immunohistochemistry, consistent with current diagnostic standards.

## Conclusion

This case highlights that MPM should be considered in the differential diagnosis of pleural effusion in young adults, even in the absence of asbestos exposure. Early pleural biopsy with immunohistochemical evaluation is essential to avoid misdiagnosis, particularly in TB-endemic settings. Although genetic testing could not be performed, this case underscores the growing relevance of genetic susceptibility in young-onset mesothelioma and the importance of clinical awareness to facilitate timely diagnosis and management in resource-limited regions.

## Data Availability

All data supporting the findings of this report are available within the article.
